# Association between Indices of Body Composition and Abnormal Metabolic Phenotype in Normal-Weight Chinese Adults

**DOI:** 10.3390/ijerph14040391

**Published:** 2017-04-07

**Authors:** Lili Xia, Fen Dong, Haiying Gong, Guodong Xu, Ke Wang, Fen Liu, Li Pan, Ling Zhang, Yuxiang Yan, Herbert Gaisano, Yan He, Guangliang Shan

**Affiliations:** 1 Department of Epidemiology and Biostatistics, School of Public Health, Capital Medical University, No. 10, Xitoutiao, You’anmen Wai, Fengtai District, Beijing 100069, China; passport2@126.com (L.X.); liufen05@ccmu.edu.cn (F.L.); zlilyepi@ccmu.edu.cn (L.Z.); Yanyx100@163.com (Y.Y.); 2 China-Japan Friendship Hospital, Cherry Garden East Street, Chaoyang District, Beijing 100029, China; fionarab@163.com (F.D.); xgdfirst@163.com (G.X.); 3 Department of Chronic Disease, Beijing Fangshan District Center for Disease Prevention and Control, Yuehua North Street, Fangshan District, Beijing 102446, China; gonghaiyinglb@126.com; 4 Birth Defects Monitoring Center, West China Second University Hospital, People South Road, Wuhou District, Chengdu 610041, China; wangkehope@126.com; 5 Institute of Basic Medical Sciences, Chinese Academy of Medical Sciences, No. 3, Dongdan, Dongcheng District, Beijing 100005, China; panli1716@163.com; 6 Departments of Medicine and Physiology, University of Toronto, Toronto, ON M5S 1A8, Canada; herbert.gaisano@utoronto.ca

**Keywords:** body composition, body fat, skeletal muscle, metabolism, metabolically unhealthy normal-weight

## Abstract

We aimed to determine the association of indices of body composition with abnormal metabolic phenotype, and to examine whether the strength of association was differentially distributed in different age groups in normal-weight Chinese adults. A total of 3015 normal-weight adults from a survey of Chinese people encompassing health and basic physiological parameters was included in this cross-sectional study. We investigated the association of body composition measured by bioelectrical impedance analysis and conventional body indices with metabolically unhealthy normal-weight (MUHNW) adults, divided by age groups and gender. Associations were assessed by multiple logistic regression analysis. We found abnormal metabolism in lean Chinese adults to be associated with higher adiposity indices (body mass index, BMI), waist circumference, and percentage body fat), lower skeletal muscle %, and body water %. Body composition was differentially distributed in age groups within the metabolically healthy normal weight (MHNW)/MUHNW groups. The impact of factors related to MUHNW shows a decreasing trend with advancing age in females and disparities of factors (BMI, body fat %, skeletal muscle %, and body water %) associated with the MUHNW phenotype in the elderly was noticed. Those factors remained unchanged in males throughout the age range, while the association of BMI, body fat %, skeletal muscle %, and body water % to MUHNW attenuated and grip strength emerged as a protective factor in elderly females. These results suggest that increased adiposity and decreased skeletal muscle mass are associated with unfavorable metabolic traits in normal-weight Chinese adults, and that MUHNW is independent of BMI, while increased waist circumference appears to be indicative of an abnormal metabolic phenotype in elderly females.

## 1. Introduction

The fact that obesity is a confirmed risk factor for chronic diseases including type 2 diabetes (T2DM) and cardiovascular diseases (CVD) has made it a major global health threat [1,2]. Body mass index (BMI) is the most commonly acknowledged diagnostic measurement for obesity. Nevertheless, individuals that are within a normal BMI range are not necessarily immune to metabolic disorders that are typically associated with obesity [3,4,5,6].

Lean individuals with abnormal metabolic profiles like hyperglycemia, hypertension, and dyslipidemia have been defined as “metabolically unhealthy normal-weight” (MUHNW), a concept first introduced by Ruderman et al. over 30 years ago [3]. MUHNW individuals may also be predisposed to similar adverse health outcomes as those observed with obese patients. However, since they are not overweight or obese, they may not get adequate medical attention, thus increasing their risk for untreated complications, which has been supported by a limited number of studies. A Korean study showed MUHNW adults exhibited increased arterial stiffness and carotid atherosclerosis compared with metabolically healthy obese adults [7]. MUHNW adults were three-fold more likely to develop diabetes than metabolically healthy obese individuals; and both groups had a similar risk in developing subclinical atherosclerosis as metabolically unhealthy obese adults [8,9]. Surprisingly, the highest risk of CVD and all-cause mortality of MUHNW adults was observed in a prospective cohort study with a median follow-up of ten years [6].

There have been several studies that attempted to estimate the prevalence of the MUHNW phenotype in the general population. Because of different criteria used to define MUHNW, the prevalence can be quite heterogeneous, ranging from 2.2% to 53.6% [10]. Even when defined by at least two metabolic abnormalities, the prevalence of MUHNW still differed in various population studies. For example, in a Korean study of the general population aged over 20 years old, MUHNW individuals accounted for 13% of the general population and 18.6% among normal-weight individuals, while in Americans of the same age range the proportions were 8.1% and 23.5%, respectively [11,12]. People with this phenotype have some common characteristics, including older age groups, alcohol drinker, smoker, and having a sedentary lifestyle [10,11,12].

No study has investigated the abnormal metabolic phenotypes assessed by the indices of body composition in a population-based study with a large sample size. Of particular note are those individuals predisposed to adiposity but with relatively low BMI, which is typical of the Asian population [13,14]; such a study is well suited to be performed in the Chinese population. The results from this study will determine the prevalence of the abnormal metabolic phenotype in the normal-weight population, and provide the rationale to propose earlier prevention of metabolic diseases in this medically neglected population. Since body composition varies with gender and age [15,16], this study has added value in assessing the specific association of abnormal metabolic phenotypes and body composition stratified into different gender and age groups.

## 2. Materials and Methods

### 2.1. Study Population

An ongoing population-based survey of Chinese people encompassing health and basic physiological parameters was conducted from 2013 onwards, which covered five provinces, including Hainan, Shanxi, Qinghai, Gansu, and Jiangxi. A stratified multistage, random cluster process was employed to select subjects. This study was approved by the Institute of Basic Medical Sciences, Chinese Academy of Medical Sciences (approval number 029-2013). Written consent was obtained from all participants. 

The data of the present study was from the site of the Shanxi province. The sampling method for this site was: first, four administrative regions were randomly sampled from the 104 districts in the Shanxi province, with two located in urban areas, one in a suburb area and one in a rural area, respectively. After that, six residential communities and six natural villages in those four regions were randomly chosen, based on a list provided by the Center for Disease Control of the Shanxi province. A total of 6410 residents aged 7–79 years old who lived in the selected communities were involved in this study. Among the 6410 participants, according to the results of a previous meta-analysis in the Chinese population [17], 681 (298 males, 383 females) were underweight (BMI < 18.5), 3220 (1248 males, 1972 females) were normal-weight (BMI 18.5–23.9), 1894 (902 males, 992 females) were overweight (BMI 24.0–27.9), and 1894 (902 males, 992 females) were obese (BMI ≥ 28). Exclusion criteria included those aged below 18 years (*n* = 588), BMI < 18.5 (*n* = 681), BMI ≥ 24 (*n* = 2509), and those with incomplete information to define the metabolic phenotype (n = 435). This left a total of 3015 normal-weight adults aged 18–79 years that were included in this study.

### 2.2. Data Collection

The information of demographic and socioeconomic status, smoking, and drinking status were obtained from participants using a questionnaire. Smoking status was divided into two categories: current smoker, or non-current smoker, according to the answers to “Are you currently a smoker”. Drinking status was categorized as current drinker and non-current drinker in the same way. Educational attainments were classified into three groups: ≤ middle school, high school, and ≥ college. Body compositions (fat mass, total body water, muscle mass), blood glucose and lipid profile, uric acid, alanine transaminase, and aspartate transaminase were collected. The latter three were chosen because they have been associated with insulin resistance in the liver [18] and overall metabolism [19,20].

Weight was measured to the nearest 0.1 kg using a SECA 813 digital scale (Seca, Vogel & Halke GmbH & Co., Hamburg, Germany), with individuals wearing only light underwear and after emptying the bladder. Body height was measured to the nearest 0.1 cm using a flexible anthropometer [21]. Waist circumference (WC) was measured at the midpoint between the lower border of the rib cage and the iliac crest. Blood pressure (BP) was measured in triplicate after a 10-min rest, using an Omron electronic sphygmomanometer (Omron Healthcare Co. Ltd., Kyoto, Japan). Handgrip was measured using a hand-held Takei dynamometer (Takei Scientific Instruments Co. Ltd., Niigata, Japan) in a standing position with the arm extended straight down to the side, and participants were asked to measure twice with their dominant hand. The larger reading of the two measurements was recorded as handgrip strength and expressed in kilograms.

Body fat mass, total body water, and impedance were measured by bioelectrical impedance (BI) analysis, which was performed with a TANITA body composition analyzer 420 (TANITA Co., Tokyo, Japan). BMI was calculated as weight (kg) divided by height squared (m^2^). Skeletal muscle mass (kg) was estimated via the following BI analysis equation of Janssen et al. [22]:

Skeletal muscle mass = (0.401 × (*height^2^*/*BI*) + (3.825 × *sex*) + (−0.071 × *age*)) + 5.102 (1)

With *height* measured in centimeters, *BI* measured in ohms, *sex* coded 1 for men and 0 for women, and *age* measured in years. The percentage of body fat, body water, and skeletal muscle were calculated as the according mass divided by the body weight.

Venous blood samples were obtained after an overnight fast of at least 8 h. All blood samples were analyzed in a national central laboratory in Beijing using the Olympus auto-analyzer 2700 (Olympus Instruments Inc., Tokyo, Japan), with strict quality control. Fasting glucose was measured by the glucose oxidase method (GOD-PAP) method (Randox Laboratories Ltd., Crumlin, UK). Serum triglyceride (TG) was measured by the glycerol lipase oxidase (GPO-PAP) method (Kyowa Medex Co. Ltd., Tokyo, Japan). Low-density lipoprotein cholesterol (LDLC) and high-density lipoprotein cholesterol (HDL-C) concentrations were measured enzymatically (Kyowa Medex Co. Ltd., Tokyo, Japan). Alanine transaminase (ALT) and aspartate transaminase (AST) were measured enzymatically (Randox Laboratories Ltd., Crumlin, UK). Serum uric acid (UA) was measured by the enzymatic colorimetric method (Randox Laboratories Ltd., Crumlin, UK).

### 2.3. Definitions of Metabolic Phenotypes

We used the criteria established by the National Cholesterol Education Program Adult Treatment Panel III (ATP III) to identify the MUHNW phenotype [23]. The presence of one or more of the following components: (1) high blood pressure (systolic blood pressure ≥ 130 or diastolic blood pressure ≥ 85 mmHg or known treatment for hypertension); (2) hypertriglyceridemia (fasting plasma triglycerides ≥ 1.69 mmol/L); (3) low HDL cholesterol (< 1.29 mmol/L); (4) hyperglycemia (fasting plasma glucose ≥ 5.6 mmol/L or known treatment for diabetes) was defined as metabolically unhealthy. The participants were classified into two groups according to the definition: metabolically healthy normal weight (MHNW) and MUHNW.

### 2.4. Statistical Analysis

All statistical analyses were conducted using the SPSS software version 18.0 for Windows (SPSS Inc., Chicago, IL, USA). The data was expressed as mean ± standard deviation (SD) for continuous variables and number (percentage) for categorical variables, respectively. Independent *t*-tests or ANOVA (analysis of variance) was used to compare the continuous variables between groups, and the chi-square test was used for the comparison of categorical variables. Factors associated with the unhealthy metabolic phenotype were evaluated by an unconditional logistic regression analysis, in which age, education, smoking, and drinking status were adjusted. Associations between body measurements and unhealthy metabolic phenotype within different age groups were also assessed in the logistic regression model, in which continuous variables were transformed to x’ = (x − mean)/SD to standardize OR (odds ratio). All tests were two-sided, and a value of *p* < 0.05 was considered as significant.

## 3. Results

### 3.1. General Characteristics of the Study Population

The 3015 normal weight adults had a mean age of 45.5 (ranged 18–79 years), with a sex distribution of 1158 (38.4%) males and 1857 (61.6%) females. Compared with females, males were older, more likely to be current smokers and drinkers, had less body fat and more muscle, and had less favorable metabolic profiles (Table 1). Fifty-one percent (*n* = 1539) of the subjects met the criteria of being metabolically unhealthy, and the detailed characteristics are shown in Table 2. Compared to metabolically healthy normal-weight individuals, besides less favorable metabolic traits, those with an abnormal metabolic phenotype were also older, more likely to be less educated, had a higher level of adiposity indexes (WC, BMI, and body fat %), and a lower level of body water % and muscle indexes (including skeletal muscle % and handgrip strength).

### 3.2. Factors Associated with Abnormal Metabolic Phenotype in Normal-Weight Adults

Characteristics of the normal and abnormal metabolic phenotype are shown in Table 3 and Table 4, stratified by gender. In both genders, compared with their metabolically healthy counterparts, metabolically unhealthy individuals were older, had higher levels of adiposity indices, uric acid (UA), and ALT, and lower levels of skeletal muscle % and body water %. All these factors were associated with metabolic abnormality after adjusting for age, education, smoking, and drinking status. Although MUHNW females had higher aspartate transaminase (AST) levels and weaker handgrip strength, the difference no longer existed after adjusting for confounders.

### 3.3. Characteristics of Body Composition Within Each Age Group and Within Each Metabolic Subgroup

We further investigated the characteristics of body measurements by metabolic phenotypes within the young adults (18–44 years old), the middle aged (45–59 years old) and the elderly (above 60 years old), and within each metabolic status (Table 5). In males of all age groups, all the adiposity indices were higher, but skeletal muscle % and body water % were lower in the MUHNW groups when compared to the MHNW groups. In females, however, the difference of all body compositions but waist circumference and skeletal muscle % between the two metabolic phenotypes was not significant in the elderly, and grip strength was higher in individuals with normal metabolism only in the elderly.

Among the MNHW and MUHNW subgroups, compared with the young male adults, the middle-aged males had larger WC, higher body water %, and lower grip strength, while the elderly had higher body water % and lower grip strength. Moreover, the elderly males had lower BMI, higher body water %, and weaker grip strength than the middle-aged group. However, in females, compared with the young adults, the middle aged and the elderly both had increased adiposity indices, decreased skeletal muscle %, and grip strength. The elderly females also had increased WC and body fat %, and decreased skeletal muscle % and grip strength when compared to the middle-aged females.

### 3.4. Association of Indices of Body Composition with Abnormal Metabolic Phenotype Was Differentially Distributed in Different Age Groups

The impact of BMI and body fat % on abnormal metabolism showed an increasing trend throughout the age groups, reaching a peak in the elderly with OR values of 2.34 and 1.98, while the waist circumference increased 68%, 50%, and 128% of the risk with each SD increment in young males, the middle aged, and the elderly males, respectively. Skeletal muscle % and body water % were protective of the abnormal metabolic phenotype in different age stages after adjusting for confounders in males (Table 6). In females, waist circumference was a risk factor of MUHNW in all age stages, posing the strongest impact (OR (95% confidence interval (CI): 1.83 (1.48–2.28)) in the middle aged, while the impact of BMI, body fat %, skeletal muscle %, and body water % seemed to attenuate in different age stages. Surprisingly, grip strength became a protective factor in the elderly females, decreasing 32% of the risk of being metabolically unhealthy (Table 6).

## 4. Discussion

To the best of our knowledge, the present study is the first to examine the factors associated with the abnormal metabolic phenotype in normal-weight Chinese adults of different age groups. Our findings suggest that higher adiposity indices (BMI, waist circumference, body fat %), and lower skeletal muscle % and body water % are associated with abnormal metabolism in lean Chinese adults, and that the impact of factors related to the unhealthy metabolic phenotype shows a decreasing trend with increasing age in females. Of note, there are disparities in the factors associated with the MUHNW phenotype in males and females aged over 60 years. Those factors remained unchanged in males throughout the age stages, while the association of BMI, body fat %, skeletal muscle %, and body water % to MUHNW attenuated and grip strength emerged as a protective factor in the elderly female.

Our finding of the association between adiposity indices and the abnormal metabolic phenotype in normal-weight adults is well in line with previous reports. The current study and other studies of the Chinese population [24,25] have shown that BMI and WC are higher in MUHNW individuals regardless of gender, and this result is further supported by studies conducted in Korea [26,27]. Moreover, the research conducted by Dvorak et al. [28] shows that the body fat % in young women with MUHNW is higher than in normal women, which also holds true in men and women below 60 years of age in our study. However, in the Dvorak et al. and Conus et al. [5] studies, the BMI and WC in women with abnormal metabolism were not significantly different from normal women. This inconsistency might have resulted from the ethnic differences, since Asians are verified to have more visceral fat than Europeans at a given WC or BMI [14,29]. Visceral fat (VAT) accumulation is a plausible mechanism for the metabolically unhealthy phenotype in our study. VAT not only acts as a fat-deposit site, but also as a highly secretory organ with a differential production of adipokines capable of regulating energy expenditure, lipid metabolism, insulin sensitivity, and inflammation [30,31]. A wealth of clinical studies have demonstrated that free fat acid (FFA), interleukin (IL)-6, C-reactive protein (CRP), and tumor necrosis factor (TNF)-α circulate at higher concentrations in individuals with greater VAT, indicating a pro-inflammatory feature [32,33,34,35]. In addition, increased macrophage infiltration has been found in both the subcutaneous and visceral adiposity tissue of individuals with abnormal metabolism [36,37], creating a low-grade chronic inflammation which is a common etiology of obesity-related complications.

Skeletal muscle is the most abundant tissue in non-obese adults, accounting for approximately 40% of the body weight and playing a critical role in energy expenditure and glucose homeostasis [38,39]. Although the impact of skeletal muscle mass on metabolism status has been less evaluated in normal-weight adults, previous reports can provide some clues. Recently, in the Korean sarcopenic obesity study, researchers used thigh muscle cross-sectional area corrected by weight as an index of muscle mass, and found that it decreased in MUHNW [16]. Furthermore, a clinical study demonstrated that in normal-weight young adults, malfunction of skeletal muscle diverted ingested glucose to the liver, leading to increased hepatic de novo lipogenesis and hyperlipidemia [40]. Since skeletal muscle accounts for the majority of glucose disposal, we presumed that the decreased skeletal muscle % accompanied by the increased fat accumulation observed in our study may play a critical role in the pathological process of the abnormal metabolic phenotype.

Our study supports previous observations that WC serves as a better indicator of metabolic risk in the elderly than BMI. Aging is associated with substantial changes in body composition, with a gradual loss of lean mass and a shift to central fat accumulation [15,41]. In this case, invisible obesity has already occurred with a perfectly normal BMI undermining metabolic health, which can partly explain a significantly higher proportion of the abnormal metabolic phenotype in the aged individuals in our study. On the contrary, WC reflects central obesity and has been validated in many epidemiological studies to be associated with increased FFA and adipokines, higher activity of inflammation, increased oxidative stress, blunt insulin sensitivity, and increased risk of developing insulin resistance and diabetes [42,43]. The shift of lean mass to fat accumulation in the elderly combined with the innate susceptibility to visceral fat deposition may account for the observed better performance of WC in both genders, even when the BMI can no longer predict unhealthy metabolism in the elderly females of our study. However, unlike elderly females, factors associated with MUHNW remained unchanged in elderly males; and although the difference in body composition is obvious, the underling mechanism of this gender disparity needs to be further investigated in a larger sample.

The present study has several limitations. First, the cross-sectional design limited our ability to infer causality from the associations observed. Second, no standard criteria for the definition of abnormal metabolism have been established. We adopted a strict definition in which satisfying any component of ATP III was considered as metabolically unhealthy. Our results might vary with different criteria. Third, the sample size of the elderly was relatively small, thus the disparity in the elderly needs to be further verified with a larger sample size and multi-ethnicities. Despite these limitations, there are several strengths of this study. We adopted a stratified multistage, randomized sampling, ensuring the representativeness of our population, thus enhancing the credibility of our results. Furthermore, the strength of association between body composition and the metabolically unhealthy normal-weight phenotype in different age groups has scarcely been investigated.

## 5. Conclusions

Our study shows that increased adiposity indices, and reduced skeletal muscle % and body water % are associated with the abnormal metabolic phenotype in normal-weight Chinese adults; however, this association between body composition and MUHNW may change with age. Moreover, waist circumference persists as a good indicator of abnormal metabolism in the elderly, especially in females.

## Figures and Tables

**Table 1:** General characteristics of the subjects.

Variables	Total (*n* = 3015)	Male (*n* = 1158)	Female (*n* = 1857)	*p* Value
Age (years)	45.5 ± 14.1	47.3 ± 15.0	44.3 ± 13.4	<0.001
Education, *n* (%)				0.324
≤Middle school	1448 (48.0)	571 (49.3)	877 (47.2)	
High school	711 (23.6)	276 (23.8)	435 (23.4)	
≥College	856 (28.4)	311 (26.9)	545 (29.3)	
Current smokers, *n* (%)	667 (22.1)	653 (56.4)	14 (0.8)	<0.001
Current drinkers, *n* (%)	622 (20.6)	531 (45.9)	91 (4.9)	<0.001
Waist (cm)	78.8 ± 6.8	81.8 ± 6.7	76.9 ± 6.1	<0.001
BMI (kg/m^2^)	21.6 ± 1.5	21.7 ± 1.5	21.5 ± 1.5	<0.001
Body fat (%)	24.8 ± 6.6	17.2 ± 3.8	28.6 ± 3.4	<0.001
Skeletal muscle (%)	41.9 ± 5.9	47.2 ± 4.7	38.7 ± 3.9	<0.001
Body water (%)	53.1 ± 4.1	57.2 ± 2.9	50.6 ± 2.2	<0.001
Handgrip strength (kg)	28.0 ± 9.2	37.0 ± 7.1	22.3 ± 4.9	<0.001
SBP (mmHg)	116.6 ± 16.2	121.1 ± 14.8	113.8 ± 16.4	<0.001
DBP (mmHg)	72.7 ± 10.0	75.0 ± 9.9	71.2 ± 9.8	<0.001
FPG (mmol/L)	5.1 ± 1.0	5.2 ± 1.1	5.0 ± 1.0	0.002
TC (mmol/L)	4.3 ± 0.9	4.2 ± 0.8	4.3 ± 1.0	0.001
TG (mmol/L)	1.4 ± 0.9	1.5 ± 1.0	1.4 ± 0.9	<0.001
HDL-C (mmol/L)	1.3 ± 0.3	1.2 ± 0.3	1.4 ± 0.3	<0.001
LDL-C (mmol/L)	2.5 ± 0.8	2.5 ± 0.7	2.5 ± 0.8	0.660
UA (μmol/L)	276.5 ± 72.0	320.6 ± 67.5	249.0 ± 60.1	<0.001
ALT (U/L)	18.8 ± 11.0	22.3 ± 13.2	16.5 ± 8.6	<0.001
AST (U/L)	21.9 ± 7.4	23.3 ± 8.4	21.1 ± 6.5	<0.001

Data are expressed as mean ± standard distribution (SD) or number (percentage). BMI: body mass index, SBP: systolic blood pressure, DBP: diastolic blood pressure, FPG: fasting plasma glucose, TC: total cholesterol, TG: triglyceride, HDL-C: high density lipoprotein cholesterol, LDL-C: low density lipoprotein cholesterol, UA: uric acid, ALT: alanine transaminase, AST: aspartate transaminase. *p* values were from independent two-sample *t*-tests or chi-square tests.

**Table 2:** Clinical and demographic characteristics of the subjects by metabolic phenotype.

Variables	Total (*n* = 3015)	MHNW (*n* = 1476)	MUHNW (*n* = 1539)	*p* Value
Age (years)	45.5 ± 14.1	42.7 ± 13.6	48.1 ± 14.1	<0.001
Gender, *n* (%)				0.313
Male	1158 (38.4)	581 (39.4)	577 (37.5)	
Female	1857 (61.6)	895 (60.6)	962 (62.5)	
Education, *n* (%)				<0.001
≤ Middle school	1448 (48.0)	647 (43.8)	801 (52.0)	
High school	711 (23.6)	338 (22.9)	373 (24.2)	
≥ College	856 (28.4)	491 (33.3)	365 (23.7)	
Current smokers, *n* (%)	667 (22.1)	324 (22.0)	343 (22.3)	0.861
Current drinkers, *n* (%)	622 (20.6)	320 (21.7)	302 (19.6)	0.163
Waist (cm)	78.8 ± 6.8	77.1 ± 6.5	80.5 ± 6.6	<0.001
BMI (kg/m^2^)	21.6 ± 1.5	21.3 ± 1.5	21.9 ± 1.4	<0.001
Body fat (%)	24.8 ± 6.6	23.2 ± 6.7	25.2 ± 6.3	<0.001
Skeletal muscle (%)	41.9 ± 5.9	43.0 ± 6.0	40.9 ± 5.6	<0.001
Body water (%)	53.1 ± 4.1	53.6 ± 4.2	52.7 ± 4.0	<0.001
Handgrip strength (kg)	28.0 ± 9.2	28.5 ± 9.1	27.4 ± 9.3	0.001
SBP (mmHg)	116.6 ± 16.2	112.9 ± 13.5	120.2 ± 17.7	<0.001
DBP (mmHg)	72.7 ± 10.0	70.8 ± 9.0	74.5 ± 10.6	<0.001
FPG (mmol/L)	5.1 ± 1.0	4.8 ± 0.4	5.3 ± 1.3	<0.001
TC (mmol/L)	4.3 ± 0.9	4.2 ± 0.8	4.4 ± 1.0	<0.001
TG (mmol/L)	1.4 ± 0.9	1.0 ± 0.3	1.8 ± 1.2	<0.001
HDL-C (mmol/L)	1.3 ± 0.3	1.5 ± 0.3	1.2 ± 0.3	<0.001
LDL-C (mmol/L)	2.5 ± 0.8	2.4 ± 0.7	2.6 ± 0.8	<0.001
UA (μmol/L)	276.5 ± 72.0	268.3 ± 67.3	284.3 ± 75.4	<0.001
ALT (U/L)	18.8 ± 11.0	17.5 ± 9.6	20.0 ± 12.0	<0.001
AST (U/L)	21.9 ± 7.4	21.7 ± 7.2	22.2 ± 7.5	0.034

Note: Data are expressed as mean ± SD or number (percentage). MHNW: metabolically healthy normal weight, MUHNW: metabolically unhealthy normal-weight. *p* values were from independent two-sample *t*-tests or chi-square tests.

**Table 3:** Factors associated with an unhealthy metabolic phenotype in normal-weight males.

Variables	MHNW (*n* = 581)	MUHNW (*n* = 577)	*p* Value ^1^	*p* Value ^2^	OR (95% CI)
Age (years)	44.6 ± 15.2	50.0 ± 14.3	<0.001		
Education, *n* (%)			0.620		
≤ Middle school	284 (48.9)	287 (49.7)			
High school	134 (23.1)	142 (24.6)			
≥ College	163 (28.0)	148 (25.7)			
Current smokers, *n* (%)	322 (55.4)	331 (57.4)	0.515		
Current drinkers, *n* (%)	263 (45.3)	268 (46.4)	0.723		
Waist (cm)	80.1 ± 6.5	83.6 ± 6.4	<0.001	<0.001	1.09 (1.06–1.11)
BMI (kg/m^2^)	21.3 ± 1.5	22.1 ± 1.3	<0.001	<0.001	1.43 (1.31–1.55)
Body fat (%)	16.2 ± 3.8	18.3 ± 3.4	<0.001	<0.001	1.16 (1.12–1.21)
Skeletal muscle (%)	48.2 ± 4.8	46.0 ± 4.2	<0.001	<0.001	0.54 (0.46–0.64)
Body water (%)	57.7 ± 3.0	56.7 ± 2.8	<0.001	<0.001	0.84 (0.80–0.88)
Handgrip strength (kg)	37.3 ± 7.0	36.6 ± 7.2	0.099	0.282	1.01 (0.99–1.03)
UA (μmol/L)	312.5 ± 63.3	328.7 ± 70.6	<0.001	<0.001	1.01 (1.00–1.01)
ALT (U/L)	21.0 ± 12.2	23.6 ± 14.0	0.001	<0.001	1.02 (1.01–1.03)
AST (U/L)	23.4 ± 8.9	23.2 ± 7.9	0.708	0.328	0.99 (0.98–1.01)

Data are expressed as mean ± SD or number (percentage). *p* value ^1^ was calculated by an independent two-sample *t*-test or chi-square test. *p* Value ^2^ and odds ratio (OR) (95% confidence interval (CI)) were obtained from logistic regression analysis adjusted for age, education, smoking, and drinking status, and the variables were standardized before entering into the logistic regression model.

**Table 4:** Factors associated with an unhealthy metabolic phenotype in normal-weight females.

Variables	MHNW (*n* = 895)	MUHNW (*n* = 962)	*p* Value ^1^	*p* Value ^2^	OR (95% CI)
Age (years)	41.5 ± 12.3	46.9 ± 13.9	<0.001		
Education, *n* (%)			<0.001		
≤ Middle school	363 (40.6)	514 (53.4)			
High school	204 (22.8)	231 (24.0)			
≥ College	328 (36.6)	217 (22.6)			
Current smokers, *n* (%)	2 (0.2)	12 (1.2)	0.013		
Current drinkers, *n* (%)	57 (6.4)	34 (3.5)	0.005		
Waist (cm)	75.1 ± 5.8	78.6 ± 5.9	<0.001	<0.001	1.09 (1.07–1.11)
BMI (kg/m^2^)	21.2 ± 1.5	21.8 ± 1.4	<0.001	<0.001	1.22 (1.14–1.31)
Body fat (%)	27.7 ± 3.5	29.3 ± 3.1	<0.001	<0.001	1.12 (1.09–1.16)
Skeletal muscle (%)	39.6 ± 3.9	37.8 ± 3.7	<0.001	<0.001	0.56 (0.48–0.66)
Body water (%)	50.9 ± 2.2	50.2 ± 2.2	<0.001	<0.001	0.86 (0.82–0.89)
Handgrip strength (kg)	22.3 ± 4.7	21.9 ± 5.0	<0.001	0.170	0.99 (0.97–1.01)
UA (μmol/L)	239.7 ± 52.9	257.6 ± 64.9	<0.001	<0.001	1.01 (1.00–1.01)
ALT (U/L)	15.2 ± 6.6	17.8 ± 10.0	<0.001	<0.001	1.03 (1.02–1.05)
AST (U/L)	20.5 ± 5.6	21.6 ± 7.2	<0.001	0.542	1.01 (0.99–1.02)

Data are expressed as mean ± SD or number (percentage). *p* Value ^1^ was calculated by an independent two-sample *t*-test or chi-square test. *p* Value ^2^ and OR (95% CI) were obtained from logistic regression analysis adjusted for age, education, smoking, and drinking status, and variables were standardized before entering into the logistic regression model.

**Table 5:** Characteristics of body measurements in subjects by different metabolic phenotypes and age groups.

Body Measurements	≤44 Years Old		*p* Value	45–59 Years Old		*p* Value	≥ 60 Years Old		*p* Value	*p* Value ^1^	*p* Value ^2^
	MHNW (282M/500F)	MUHNW (193M/386F)		MHNW (182M/321F)	MUHNW (225M/406F)		MHNW (117M/74F)	MUHNW (159M/170F)			
Waist											
Males	79.2 ± 5.7	82.0 ± 6.1	<0.001	81.7 ± 6.1 ^a^	84.2 ± 7.0 ^e^	0.001	80.3 ± 8.2	84.7 ± 5.6 ^d^	<0.001	0.003	0.002
Females	73.7 ± 5.5	76.3 ± 5.4	<0.001	76.4 ± 5.2 ^a^	79.7 ± 5.7 ^d^	<0.001	79.3 ± 6.5 ^a,b^	81.3 ± 5.9 ^d,g^	0.030	<0.001	<0.001
BMI											
Males	21.4 ± 1.5	22.0 ± 1.5	<0.001	21.8 ± 1.5	22.3 ± 1.2	<0.001	21.3 ± 1.5 ^c^	22.2 ± 1.2	<0.001	0.099	0.222
Females	20.8 ± 1.4	21.4 ± 1.5	<0.001	21.4 ± 1.4 ^a^	21.9 ± 1.4 ^d^	<0.001	21.9 ± 1.4 ^a^	22.0 ± 1.4 ^d^	0.176	<0.001	<0.001
Body fat %											
Males	16.2 ± 3.8	18.0 ± 3.6	<0.001	17.2 ± 3.8	18.8 ± 3.3	<0.001	16.7 ± 3.4	18.4 ± 3.4	<0.001	0.220	0.070
Females	26.8 ± 3.6	28.4 ± 3.1	<0.001	28.4 ± 3.0 ^a^	29.6 ± 2.9 ^d^	<0.001	29.6 ± 3.0 ^a,c^	30.3 ± 3.0 ^d,g^	0.083	<0.001	<0.001
Skeletal muscle %											
Males	48.3 ± 5.2	46.3 ± 5.2	<0.001	47.9 ± 4.5	45.5 ± 3.9 ^e^	<0.001	48.2 ± 4.4	46.4 ± 4.4 ^g^	0.001	0.644	0.046
Females	40.7 ± 3.8	39.1 ± 3.5	<0.001	38.5 ± 3.6 ^a^	37.3 ± 3.5 ^d^	<0.001	37.0 ± 3.0 ^a,b^	36.0 ± 3.6 ^d,f^	0.049	<0.001	<0.001
Body water %											
Males	56.7 ± 3.1	55.6 ± 2.5	<0.001	57.5 ± 2.8 ^a^	56.5 ± 2.5 ^d^	<0.001	58.7 ± 2.3 ^a,b^	57.8 ± 2.8 ^d,f^	0.001	<0.001	<0.001
Females	50.9 ± 2.4	49.9 ± 2.2	<0.001	50.9 ± 2.1	50.2 ± 2.1 ^e^	<0.001	51.2 ± 1.9	50.8 ± 2.2 ^d,f^	0.198	0.377	<0.001
Grip strength											
Males	40.6 ± 5.8	40.8 ± 5.7	0.410	36.9 ± 6.2 ^a^	37.3 ± 6.2 ^d^	0.708	32.8 ± 7.1 ^a,b^	33.1 ± 6.9 ^d,f^	0.928	<0.001	<0.001
Females	23.8 ± 4.5	23.7 ± 4.6	0.644	22.1 ± 4.8 ^a^	22.0 ± 4.4 ^d^	0.777	20.5 ± 4.4 ^a,b^	18.1 ± 3.8 ^d,f^	0.002	<0.001	<0.001

Data are expressed as mean ± SD. *p* values were calculated by an independent two-sample *t*-test. *p* value ^1^ and *p* value ^2^ were calculated by analysis of variance (ANOVA) within MHNW and MUHNW groups across the age groups, respectively. Comparisons between two different age groups within metabolic subgroups were analyzed by the least significant difference (LSD) post hoc test; ^a^ *p* < 0.001 vs. MHNW in young adults; ^b^ *p* < 0.001 vs. MHNW in the middle aged adults; ^c^ *p* < 0.05 vs. MHNW in the middle aged adults; ^d^ *p* < 0.001 vs. MUHNW in young adults; ^e^ *p* < 0.05 vs. MUHNW in young adults; ^f^ *p* < 0.001 vs. MUHNW in the middle aged adults; ^g^ *p* < 0.05 vs. MUHNW in the middle aged adults.

**Table 6:** Association between body measurements and abnormal metabolic phenotype in normal-weight adults by age groups.

Body Measurements	≤44 Years Old		45–59 Years Old		≥60 Years Old	
	OR (95% CI)	*p* Value	OR (95% CI)	*p* Value	OR (95% CI)	*p* Value
Waist						
Males	1.68 (1.29–2.17)	<0.001	1.50 (1.13–2.00)	0.006	2.28 (1.56–3.31)	<0.001
Females	1.67 (1.40–2.00)	<0.001	1.83 (1.48–2.28)	<0.001	1.45 (1.03–2.04)	0.032
BMI						
Males	1.55 (1.28–1.87)	<0.001	1.61 (1.28–2.01)	<0.001	2.34 (1.74–3.15)	<0.001
Females	1.45 (1.26–1.67)	<0.001	1.36 (1.15–1.60)	<0.001	1.28 (0.95–1.73)	0.108
Body fat %						
Males	1.65 (1.34–2.03)	<0.001	1.87 (1.46–2.41)	<0.001	1.98 (1.48–2.65)	<0.001
Females	1.57 (1.35–1.82)	<0.001	1.47 (1.23–1.75)	<0.001	1.35 (0.99–1.85)	0.062
Skeletal muscle %						
Males	0.63 (0.48–0.82)	0.001	0.45 (0.33–0.61)	<0.001	0.54 (0.38–0.76)	0.001
Females	0.51 (0.41–0.65)	<0.001	0.61 (0.47–0.79)	<0.001	0.65 (0.40–1.07)	0.089
Body water %						
Males	0.64 (0.51–0.79)	<0.001	0.54 (0.42–0.70)	<0.001	0.61 (0.46–0.81)	0.001
Females	0.68 (0.59–0.78)	<0.001	0.72 (0.61–0.84)	<0.001	0.75 (0.56–1.01)	0.060
Grip strength						
Males	1.07 (0.86–1.35)	0.539	1.08 (0.86–1.37)	0.546	1.08 (0.82–1.41)	0.591
Females	0.95 (0.83–1.10)	0.485	1.08 (0.92–1.28)	0.362	0.68 (0.47–0.97)	0.035

*p* Value and OR (95% CI) were obtained from logistic regression analysis adjusted for age, education, smoking, and drinking status. Variables were standardized before entering into logistic regression mode.
